# 155. *In Vitro* Evaluation of Delafloxacin Activity Against Contemporary US Isolates from Community-Acquired Pneumonia and Community-Acquired Lower Respiratory Tract Infections: Results from the SENTRY Antimicrobial Surveillance Program (2014-2020)

**DOI:** 10.1093/ofid/ofab466.155

**Published:** 2021-12-04

**Authors:** Dee Shortridge, Jennifer M Streit, Michael D Huband, Mariana Castanheira, Mariana Castanheira

**Affiliations:** 1 JMI Labortories, North Liberty, Iowa; 2 JMI Laboratories, North Liberty, IA

## Abstract

**Background:**

Delafloxacin (DLX) is a broad-spectrum fluoroquinolone antibacterial approved in the US for the treatment of community-acquired bacterial pneumonia (CABP). DLX is indicated to treat CABP caused by *Streptococcus pneumoniae* (SPN), *Haemophilus influenzae* (HI), *Haemophilus parainfluenzae* (HP), methicillin-susceptible *Staphylococcus aureus* (MSSA), *Escherichia coli* (EC), *Klebsiella pneumoniae* (KPN), *Pseudomonas aeruginosa* (PSA), *Chlamydia pneumoniae*, *Mycoplasma pneumoniae* and *L. pneumophila*. In this study, the *in vitro* susceptibilities of DLX and comparator quinolones were determined for clinical isolates from CAP and CA-lower respiratory tract infections (LRTIs).

**Methods:**

CAP and CA-LRTI isolates were consecutively collected at 67 US medical centers participating in the SENTRY Surveillance Program during 2014-2020. Sites submitted 1 isolate per patient per infection episode. Isolate identification was determined at each site and confirmed using standard biochemical or molecular methods at JMI Laboratories. Susceptibility testing was performed according to CLSI broth microdilution methodology. CLSI (2021) interpretive criteria were applied, FDA criteria were used for DLX.

**Results:**

The susceptibility results for DLX, levofloxacin (LEV), moxifloxacin (MOX), and ciprofloxacin (CIP) for the indicated species are shown in the table. As MOX does not have CLSI breakpoints for EC, KPN, or PSA, CIP was tested for those species instead. DLX had the highest percent susceptibility against MSSA (91.8%). SPN and HI were >97% susceptible, and HP was >91% for all 3 drugs. KPN susceptibility ranged from 86.4% for LEV to 76.9% for DLX. Susceptibilities for EC and PSA were similar for the 3 drugs, EC varied from 59.8% for LEV to 57.0% for DLX, and PSA varied from 71.6% for CIP to 64.0% to LEV.

**Conclusion:**

DLX had good activity against recent CAP and CA-LRTI isolates from US hospitals. DLX had the highest susceptibility of the quinolones tested against MSSA. Quinolone-resistant SPN and HI were uncommon. These *in vitro* results suggest that DLX may be a useful therapeutic option for CABP caused by Gram-positive, Gram-negative and fastidious pathogens.

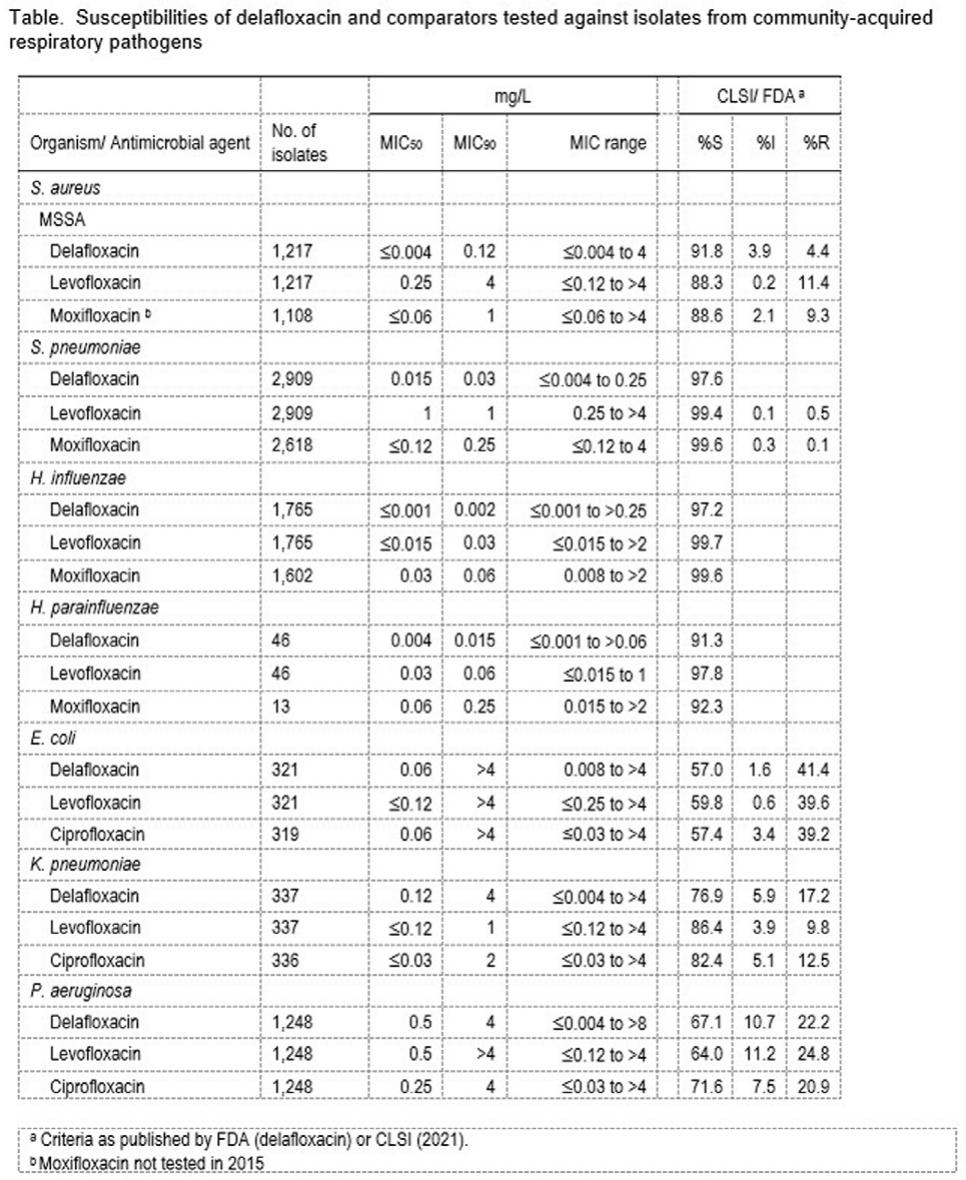

**Disclosures:**

**Dee Shortridge, PhD**, **AbbVie (formerly Allergan**) (Research Grant or Support)**Melinta Therapeutics, Inc.** (Research Grant or Support)**Melinta Therapeutics, LLC** (Research Grant or Support)**Shionogi** (Research Grant or Support) **Jennifer M. Streit, BS**, **GlaxoSmithKline, LLC** (Research Grant or Support)**Melinta Therapeutics, LLC** (Research Grant or Support)**Shionogi** (Research Grant or Support)**Spero Therapeutics** (Research Grant or Support) **Michael D. Huband, BS**, **AbbVie (formerly Allergan**) (Research Grant or Support)**Melinta Therapeutics, LLC** (Research Grant or Support) **Mariana Castanheira, PhD**, **AbbVie (formerly Allergan**) (Research Grant or Support)**Bravos Biosciences** (Research Grant or Support)**Cidara Therapeutics, Inc.** (Research Grant or Support)**Cipla Therapeutics** (Research Grant or Support)**Cipla USA Inc.** (Research Grant or Support)**GlaxoSmithKline** (Research Grant or Support)**Melinta Therapeutics, Inc.** (Research Grant or Support)**Melinta Therapeutics, LLC** (Research Grant or Support)**Pfizer, Inc.** (Research Grant or Support)**Qpex Biopharma** (Research Grant or Support)**Shionogi** (Research Grant or Support)**Spero Therapeutics** (Research Grant or Support) **Mariana Castanheira, PhD**, Affinity Biosensors (Individual(s) Involved: Self): Research Grant or Support; Allergan (Individual(s) Involved: Self): Research Grant or Support; Amicrobe, Inc (Individual(s) Involved: Self): Research Grant or Support; Amplyx Pharma (Individual(s) Involved: Self): Research Grant or Support; Artugen Therapeutics USA, Inc. (Individual(s) Involved: Self): Research Grant or Support; Astellas (Individual(s) Involved: Self): Research Grant or Support; Basilea (Individual(s) Involved: Self): Research Grant or Support; Beth Israel Deaconess Medical Center (Individual(s) Involved: Self): Research Grant or Support; BIDMC (Individual(s) Involved: Self): Research Grant or Support; bioMerieux Inc. (Individual(s) Involved: Self): Research Grant or Support; BioVersys Ag (Individual(s) Involved: Self): Research Grant or Support; Bugworks (Individual(s) Involved: Self): Research Grant or Support; Cidara (Individual(s) Involved: Self): Research Grant or Support; Cipla (Individual(s) Involved: Self): Research Grant or Support; Contrafect (Individual(s) Involved: Self): Research Grant or Support; Cormedix (Individual(s) Involved: Self): Research Grant or Support; Crestone, Inc. (Individual(s) Involved: Self): Research Grant or Support; Curza (Individual(s) Involved: Self): Research Grant or Support; CXC7 (Individual(s) Involved: Self): Research Grant or Support; Entasis (Individual(s) Involved: Self): Research Grant or Support; Fedora Pharmaceutical (Individual(s) Involved: Self): Research Grant or Support; Fimbrion Therapeutics (Individual(s) Involved: Self): Research Grant or Support; Fox Chase (Individual(s) Involved: Self): Research Grant or Support; GlaxoSmithKline (Individual(s) Involved: Self): Research Grant or Support; Guardian Therapeutics (Individual(s) Involved: Self): Research Grant or Support; Hardy Diagnostics (Individual(s) Involved: Self): Research Grant or Support; IHMA (Individual(s) Involved: Self): Research Grant or Support; Janssen Research & Development (Individual(s) Involved: Self): Research Grant or Support; Johnson & Johnson (Individual(s) Involved: Self): Research Grant or Support; Kaleido Biosceinces (Individual(s) Involved: Self): Research Grant or Support; KBP Biosciences (Individual(s) Involved: Self): Research Grant or Support; Luminex (Individual(s) Involved: Self): Research Grant or Support; Matrivax (Individual(s) Involved: Self): Research Grant or Support; Mayo Clinic (Individual(s) Involved: Self): Research Grant or Support; Medpace (Individual(s) Involved: Self): Research Grant or Support; Meiji Seika Pharma Co., Ltd. (Individual(s) Involved: Self): Research Grant or Support; Melinta (Individual(s) Involved: Self): Research Grant or Support; Menarini (Individual(s) Involved: Self): Research Grant or Support; Merck (Individual(s) Involved: Self): Research Grant or Support; Meridian Bioscience Inc. (Individual(s) Involved: Self): Research Grant or Support; Micromyx (Individual(s) Involved: Self): Research Grant or Support; MicuRx (Individual(s) Involved: Self): Research Grant or Support; N8 Medical (Individual(s) Involved: Self): Research Grant or Support; Nabriva (Individual(s) Involved: Self): Research Grant or Support; National Institutes of Health (Individual(s) Involved: Self): Research Grant or Support; National University of Singapore (Individual(s) Involved: Self): Research Grant or Support; North Bristol NHS Trust (Individual(s) Involved: Self): Research Grant or Support; Novome Biotechnologies (Individual(s) Involved: Self): Research Grant or Support; Paratek (Individual(s) Involved: Self): Research Grant or Support; Pfizer (Individual(s) Involved: Self): Research Grant or Support; Prokaryotics Inc. (Individual(s) Involved: Self): Research Grant or Support; QPEX Biopharma (Individual(s) Involved: Self): Research Grant or Support; Rhode Island Hospital (Individual(s) Involved: Self): Research Grant or Support; RIHML (Individual(s) Involved: Self): Research Grant or Support; Roche (Individual(s) Involved: Self): Research Grant or Support; Roivant (Individual(s) Involved: Self): Research Grant or Support; Salvat (Individual(s) Involved: Self): Research Grant or Support; Scynexis (Individual(s) Involved: Self): Research Grant or Support; SeLux Diagnostics (Individual(s) Involved: Self): Research Grant or Support; Shionogi (Individual(s) Involved: Self): Research Grant or Support; Specific Diagnostics (Individual(s) Involved: Self): Research Grant or Support; Spero (Individual(s) Involved: Self): Research Grant or Support; SuperTrans Medical LT (Individual(s) Involved: Self): Research Grant or Support; T2 Biosystems (Individual(s) Involved: Self): Research Grant or Support; The University of Queensland (Individual(s) Involved: Self): Research Grant or Support; Thermo Fisher Scientific (Individual(s) Involved: Self): Research Grant or Support; Tufts Medical Center (Individual(s) Involved: Self): Research Grant or Support; Universite de Sherbrooke (Individual(s) Involved: Self): Research Grant or Support; University of Iowa (Individual(s) Involved: Self): Research Grant or Support; University of Iowa Hospitals and Clinics (Individual(s) Involved: Self): Research Grant or Support; University of Wisconsin (Individual(s) Involved: Self): Research Grant or Support; UNT System College of Pharmacy (Individual(s) Involved: Self): Research Grant or Support; URMC (Individual(s) Involved: Self): Research Grant or Support; UT Southwestern (Individual(s) Involved: Self): Research Grant or Support; VenatoRx (Individual(s) Involved: Self): Research Grant or Support; Viosera Therapeutics (Individual(s) Involved: Self): Research Grant or Support; Wayne State University (Individual(s) Involved: Self): Research Grant or Support

